# Residential Proximity to Agricultural Fields and Neurological and Mental Health Outcomes in Rural Adults in Matlab, Bangladesh

**DOI:** 10.3390/ijerph16183228

**Published:** 2019-09-04

**Authors:** Khalid M. Khan, Janesh Karnati, Ipsita Hamid, David Koceja, Mohammad Zahirul Islam, Md Alfazal Khan

**Affiliations:** 1 Department of Environmental and Occupational Health, School of Public Health, Indiana University-Bloomington, Bloomington, IN 47405, USA; 2 Cardiovascular Research Center, Brown University, Providence, RI 02912, USA; 3 Department of Kinesiology, School of Public Health, Indiana University-Bloomington, Bloomington, IN 47405, USA; 4 International Centre for Diarrhoeal Disease Research, Mohakhali, Dhaka 1213, Bangladesh; 5 Matlab Health Research Centre, International Centre for Diarrhoeal Disease Research (icddr,b), Dhaka 1000, Bangladesh

**Keywords:** neurological outcome, pesticide exposure, residential proximity, rural Bangladesh

## Abstract

Pesticide exposure is an important rural public health concern that is linked to a spectrum of health outcomes in farmers. However, little is known about these effects on residents living in close proximity to agricultural fields and who are not involved in regular farming. This paper compared the effects of residential proximity to farming lands on a number of neurological and mental health outcomes in adults. A cross-sectional study was performed on 57 adults involved in farming only occasionally in rural Matlab in Bangladesh. A health and demographic surveillance system (HDSS) and geocoding were used to define proximity to the agricultural field. Neurological health was measured using the trail making test, vibrotactile threshold measurement, and dominant ulnar nerve conduction velocity (NCV) amplitude. An adapted Center for Epidemiological Studies Depression scale (CES-D) questionnaire was used to evaluate mental health. Results indicated that respondents living near agricultural fields had significantly higher vibrotactile threshold in big toes (*p* < 0.004) and needed a longer time to complete the trail making test (*p* < 0.004) than those living far from fields after accounting for the covariates. Results of this pilot study suggest further investigations to establish the impact of pesticide exposure among occasional and non-farmers on neurological health outcomes.

## 1. Introduction

Pesticide exposure is an important rural public health issue in many agriculture-dependent developing countries. Lack of knowledge about agricultural safety practices, especially about personal protective equipment (PPE), poses millions of rural people at greater risk to pesticide exposure. Studies conducted around the world have reported several unsafe behaviors of the pesticide applicators. In the South Asian region, where agriculture is a key determinant of economy and livelihood, the magnitude of risk of pesticide exposure is anticipated to be even higher due to a lack of resources, awareness, and education materials on PPE. A study of over 500 farmers in rural Bangladesh reported a high proportion of farmers using toxic pesticides and almost 70% did not use any PPE during application [1]. Data of this Bangladesh study was consistent with other agricultural health studies conducted elsewhere in Asia. For instance, a study in Kuwait found that over 70% of farmers did not read the pesticide application instructions, and 58% did not use any PPE when handling pesticides [2,3].

Pesticides are associated with a plethora of dangerous health effects in adult and children living in the agricultural communities. These health effects include: dermatological, gastrointestinal, neurological, carcinogenic, respiratory, reproductive, and endocrine effects [4]. Additionally, high levels of exposure can lead to hospitalization and death [5]. Though pesticides can affect the human body in various ways, the best-documented health effects of pesticide exposure involve the nervous system [6]. Pesticides can be absorbed into the human body via dermal route, ingestion or inhalation and then can affect the nervous system [4]. There are many neurotoxic consequences of exposure: deficits in neurobehavioral performance, abnormalities in nerve function, and increased risk of neurodegenerative disease [7]. Other health effects associated with pesticide exposure include: reduction in tibial nerve compound muscle action potential amplitudes, abnormal blood count, poor hepatic and renal function, and decreased nerve conduction amplitudes and velocities [7]. Additionally, it was found that pesticide exposure was linked to a higher occurrence of headache, nausea, vomiting, fatigue, and irritation of the skin and eyes [6].

In addition to the somatic symptoms and illness due to pesticide exposure, recent research has shown that exposure can also lead to mental health effects. A positive association was found between depression and occupational pesticide use among applicators. Farmers with the highest number of exposure days to pesticides were 50% more likely to have a depression diagnosis [8]. Additionally, a relationship between phenoxy herbicides and neuropsychiatric symptoms was found [9].

Although neurological effects of pesticides on low and no PPE-user farmers are well known, little is known about the effects of pesticides on non-farmers who live in agricultural communities. It has been found that children who live in close proximity to pesticide-treated farmland have a higher exposure to pesticides than others [10,11]. Another study has found that concentrations of pesticides in the air decreases as one goes further from the farmland [12]. Both of these findings support the idea of pesticide drift and suggest that those who live close to pesticide treated land will have greater pesticide exposure than those who do not. However, research has not been done to examine how proximity affects the nervous system. To address the gaps of knowledge regarding the effects of proximity to agricultural fields on the nervous system functions of rural residents, we conducted a small cross-sectional study in a sample of rural adults in Matlab, Bangladesh. We hypothesized that adults living at least 10 years or longer in close proximity (e.g., 200 m or less) to agricultural lands would have poorer performance in neurological and mental health tests compared to those rural residents who live further away from the agricultural lands.

## 2. Materials and Methods

### 2.1. Study Setting

Matlab, a rural sub-district, is located approximately 50 km south of Dhaka—the capital of Bangladesh. Here, a cohort of more than 200,000 people under the Health and Demographic Surveillance System (HDSS) of the International Centre for Diarrheal Disease Research, Bangladesh (icddr,b) has been maintained since 1966 [13]. This cross-sectional study was conducted on 57 adult residents of HDSS between the period of 30 October 2016 and 31 December 2016. Any adult visiting the central Matlab hospital in HDSS area was approached for participating in the study if he or she met the inclusion criteria such as age between 30 and 55 years, being a resident of an agricultural community in Matlab, married, physically active, non-smoker, not involved in a full-time agricultural work in the past (i.e., agricultural is not the primary occupation), and healthy without any known chronic disease. Seventy adults who met these criteria were identified and enrolled in the study by our local project team, which was composed of a lead field coordinator, a physician trained on various neurological tests, and a research assistant. Among the 70 adults who were initially approached, 57 agreed to participate in the study and subsequently made appointments to visit Matlab hospital for interviews followed by various neurological and mental health assessments. This study was approved by the Indiana University Institutional Review Board (IRB), the Ethical Review Committee (ERC) and the Research Review Committee (RRC) of icddr,b. Signed informed consent was obtained from the participant prior to visiting Matlab hospital for this study.

### 2.2. Procedure and Exposure Assessment

Prior to the hospital visit, the field coordinator and a field research assistant visited the houses of the participants to collect information about the proximity to nearby agricultural fields. Residential proximity to the nearest agricultural field was used as a proxy measure for participant’s exposure to pesticides. The unique residential address of each participant and the nearest agricultural field where pesticide applications occurred each year were physically verified and then geocoded into latitude and longitude coordinates using Google Earth (Mountain View, CA, USA). Finally, these coordinates were used to determine the straight-line distance (in meters) from each residence to the nearest field. Based on the results of the sensitivity analyses, residential proximity in this study was defined as <200, and ≥200 m from the nearest agricultural field. During the hospital visit, the height, weight, and blood pressure of each participant were measured. Each participant then attended a structured questionnaire-based interview session to provide information about sociodemographic characteristics such as household income, house and land ownership, primary and secondary occupations, and number of family members in the household. Our field staff also visited the local market to document the most commonly used pesticides available in the market during the study. After a short break, neurological and mental health evaluations of the participant were conducted by the trained study physician.

### 2.3. Neurological Outcomes Evaluation

For the evaluation of the function of peripheral and central nervous systems three non-invasive tests and one short survey were conducted in the following order:

(i) Trail making. The Trail Making Test is a neuropsychological test of visual attention and task switching [14]. There are two segments to this test. In part A, the participant was asked to draw lines to connect 25 encircled numbers distributed on a page. In part B, the same person was asked to connect circles but alternate between numbers and letters. Both sections were timed and the outcome score was the amount of time in seconds required to complete each part.
(ii) Vibrotactile threshold measurement. This is a useful technique for the identification of peripheral neuropathy [15]. A simple electromechanical vibrometer consisting of a controller unit and a transducer unit was used for rapid and quantitative assessment of peripheral nervous system function. The participant was asked to feel different intensities (amplitude) of the vibration when he/she touched the vibrating post of the transducer unit using their toes. The level of intensities at which the vibration was detected (or was no longer detected) by the participant were recorded for both the left and right great toes.
(iii) Measurement of ulnar motor nerve conduction velocity (NCV). The speed with which the ulnar motor nerve conducts an impulse was measured in the dominant hand following previously published methods [16,17]. In this non-invasive test, surface electromyography (EMG) electrodes were positioned on the hand. The ulnar nerve was stimulated at two points, one near the elbow (proximal point) and a second point near the wrist (distal point). The difference in latencies of the EMG responses at proximal and distal points were measured to estimate the conduction velocity of the motor nerve fibers in the ulnar nerve.
(iv) Neurological symptoms. A short-term, 25-item neurological symptom questionnaire used in a previous study [18] was translated in local language (i.e., Bangla) for the current study. The symptoms were grouped into six domains: behavioral, autonomic, cognitive, sensory, motor and non-specific temporary disability as described before [18]. The questionnaire had five response options (0–4) for each symptom ranging from “never” (scored as 0) to “everyday of the week” (scored as 4). The sum of the scores for each of the symptoms under a specific domain was calculated. Symptom scores for all domains were added to derive the total symptom score.

### 2.4. Depression Symptoms Measurement

A 16-item depression symptom questionnaire adapted from the Center for Epidemiological Studies Depression scale (CES-D) questionnaire [19] was administered verbally. CES-D has been validated and widely used in many developing countries across the world including Asian populations [20]. More recently, a Bangladesh study has also found CES-D a reliable instrument for measuring depression in adult women from a low-income population [21]. Twenty items of CES-D were divided into four subscales such as Depressive and Affect (e.g., felt depressed, fearful, lonely, sad), Well-Being (e.g., felt happy, enjoyable), Somatic (e.g., felt bothered, had restless sleep), and Inter-Personal (e.g., people were unfriendly, people disliked) as described in studies on Asian populations [20]. Four items of the CES-D questionnaire were not administered as they were found culturally inappropriate in our study population in a very similar way to what other Asian studies previously reported [20]. To facilitate verbal administration, a four-point response scale was used. Participants indicated how often they experienced each of the depression symptoms as ‘not at all’, ‘rarely’, ‘1–2 days in a week’, and ‘almost every day in a week’ (scored 0, 1, 2 and 3 respectively). The scoring of positive items (e.g., felt happy, enjoyable) was reversed so that higher scores indicated presence of more depression symptomatology. We summed item scores to generate four subscale scores, and summed subscale scores to generate a total CES-D score (ranged from 0 to 48).

### 2.5. Statistical Analysis

Since the purpose of the study is to generate preliminary data for larger future studies, we were not concerned about the power calculation. The major objective was to assess the feasibility of this study in a rural setting and to obtain signals of neurological and mental health effects in non-farmers which could be induced by close proximity to agricultural field. To indicate internal consistency of the CES-D and neurological symptom items, we calculated Cronbach’s alpha for each CES-D and neurological symptom subscales. We calculated summary statistics to describe the sample characteristics by comparing frequency (%) or means ± standard deviation between the two exposure groups (i.e., <200 m from the agricultural field and ≥200 m from the field). Chi-square test and t-test were used to detect group differences for categorical and continuous variables, respectively. Generalized Linear Models (GLM) were constructed to examine the association of residential distance to agricultural fields with means of neurological and mental health outcomes. The sociodemographic covariates selected for initial inclusion in the statistical models were selected based on the published literature linking those with neurological and mental health effects of pesticide exposure in healthy adults or documented risk factors for poorer neurological and mental health status. These covariates included age, gender, BMI, household income, educational qualification, house ownership, land ownership, and primary occupation. We examined whether these covariates changed the estimated associations between exposure and outcomes. Variables were retained in the models if there was substantial change (i.e., >10%) in the association of interest. The statistical analyses were performed using SAS 9.4 (SAS Institute, Cary, NC, USA) and IBM SPSS Statistics 25 (IBM, Armonk, NY, USA).

## 3. Results

### 3.1. Reliability of Outcome Subscales

Both CES-D and neurological symptom subscales demonstrated moderate to high reliability as revealed by the Cronbach’s alpha values. For CES-D subscales, the Cronbach’s alpha values ranged from 0.71 to 0.82 and for self-reported symptom subscales the same measure of internal consistency ranged from 0.68 to 0.84.

### 3.2. Sociodemographic Characteristics

We compared the sociodemographic characteristics of the participants who lived in close proximity to the agricultural (Ag) field (<200 m) (*n* = 38) with those living 200 m or more away from the Ag field (*n* = 19). No significant differences were found for participant’s sex, age, household income, land ownership, number of children in the family, educational qualification, primary occupation, and marital status (Table 1). Participants living in close proximity (<200 m) to the field had significantly higher BMI (26.89 vs. 21.80 kg/m^2^) than those living far from the field.

### 3.3. Pesticides Used in the Study Area

Pesticides sold in the local market, the only source of pesticides as reported by the participants during the interview are summarized in Table 2. It was observed that most of the pesticides used in agricultural fields in the study were highly or moderately hazardous according to WHO classification of pesticides [22].

### 3.4. Proximity to Agricultural Fields and Nervous System Outcomes

Table 3 shows the results of the unadjusted and adjusted analysis regarding the associations between distance from agricultural fields and neurological, neurobehavioral and mental health outcomes. Overall, the results indicated worse nervous system function in the high exposure group (compared to the low exposure group). For example, participants living in close proximity to the agricultural land had significantly higher mean vibrotactile threshold scores in both right and left big toes even after accounting for age, BMI, education, and household income. The same group, living close to the pesticide-exposed agricultural lands, also took more time in completing both formats of the trail making test after accounting for these covariates although the difference was statistically significant for the more complex trail making test B (136.13 vs. 110.80 s). No significant difference was observed between the two groups for nerve conduction velocity and various self-reported neurological symptom subscales. Similarly, none of the five mental health outcomes demonstrated a significant difference between the two exposure groups.

## 4. Discussion

In our research, we used several neurological tests as well as self-reported neurological and mental health symptom questionnaire for detecting differences between the groups representing close proximity (high chronic pesticide exposure) and far proximity (low chronic pesticide exposure) to agricultural fields. Our results found significant differences in the vibrotactile threshold test and trail-making test B mean scores between the two groups. We did not find significant differences between the groups for other neurological and mental health outcomes.

The vibrotactile threshold test and trail making test B measures peripheral nerve function and visual attention with task switching respectively. Both skills are associated with central nervous system function. Higher mean scores on the vibrotactile threshold test indicated that a greater stimulus was needed for detection of vibration by the participant, reflecting a potential sign of peripheral neuropathy. Additionally, higher mean scores on the trail-making test indicated potential cognitive impairment and overall lower executive functioning. These results may indicate possible long-term effects of passive pesticide exposure on both the peripheral and central nervous system, respectively.

In the past, researchers investigated the neurological effects of chronic pesticide exposure and found varying results. In a study of 164 organophosphate (OP) pesticide applicators and 83 unexposed controls (mean age = 34 vs. 33 years), vibrotactile score difference was not associated with pesticide exposure [23]. In a study comparing 191 OP termiticide applicators (mean age, 39 years) with their 106 unexposed friends and 83 unexposed workers, no difference was observed between applicators and either comparison group on measures of great toe Vibrotactile threshold [24]. One particular study on farmers and pesticide applicators in New York found a significant increase in mean vibration threshold sensitivity for the dominant and non-dominant hand of farmers compared to non-farmers and non-pesticide applicators, suggesting that a loss of peripheral nerve function is correlated to increased pesticide exposure [25]. Additionally, a meta-analysis study reported pesticide exposure to be associated with deficits in cognitive function (measured by various tests including trail making) [6]. Another study identified neurobehavioral deficits among workers exposed to pesticides in Egypt. Using a variety of methods for identifying neurobehavioral deficits including Trail Making Tests A & B, they found that exposed participants had significantly lower performance on various neurobehavioral tests (Similarities, Digit Symbol, Trail making part A and B, Letter Cancellation, Digit Span, and Benton Visual Retention) [26]. Using data collected during the period of 1986–1988, a retrospective study on the chronic neurological effects of acute pesticide poisoning in Nicaraguan agriculture workers demonstrated that those affected by acute pesticide poisoning performed significantly worse on five of six subtests of a World Health Organization neuropsychological test battery [27]. In addition to the studies mentioned above, many other studies have correlated chronic pesticide exposure to peripheral and central nervous system dysfunction.

Although we found differences in tests that measure neurological outcomes, we did not find any differences in psychological or mental health outcomes. Similar results have been found by other studies, however, research has found both significant and nonsignificant correlations between pesticide exposure and mental health outcomes. One study that evaluated neuropsychological effects due to chronic organophosphate use among farmers found no differences in mental health outcomes between farmers and non-farmers [28]. However, another study on the behavioral effects of occupational exposure of organophosphates in female greenhouse workers found that the workers exposed to organophosphates displayed a significant increase in depression and fatigue compared to those who were not exposed [29]. As observed by the varying results among studies, there is a lack of certainty on the psychological effects of chronic pesticide exposure.

There is an abundance of research on the effects of chronic pesticide exposure on neurological and psychological functioning, however, the vast majority of these studies have characterized groups of low and high pesticide exposure based on the length of occupational exposure. This study is novel in that we compared neurological and psychological symptoms with residential proximity to the agricultural field, a proxy of passive or indirect pesticide exposure. As described in the introduction, there is a growing body of evidence that shows the closer one lives to pesticide treated farmland, the higher the levels of pesticides surrounding and within one’s residence [10,12]. However, it has not been established whether or not the levels found in one’s residence and living area are high enough to cause neurological and/or psychological symptoms. Other studies showed that increased exposure led to a prevalence of neurological or psychological symptoms, this study showed that increased residential proximity to an agriculture field is correlated with decreased neurological functioning. This answers the question of whether or not the pesticide levels found in one’s residence and living area are high enough to cause neurological and/or psychological symptoms. Additionally, this paper provides further evidence for the idea of pesticide drift and that chronic pesticide exposure may produce adverse effects on nervous system functioning.

### Potential Limitations

Though we were able to identify certain relationships between proximity to agricultural fields and neurological outcomes, the relatively small sample size limits the extent to which this relationship can be used to confirm our hypothesis. However, our study findings, though limited by the small sample size, justify the need for a larger study to further explore the relationship between agricultural proximity and neurological outcomes. The current findings lay the foundation upon which a larger study with greater statistical power can be conducted.

There were further methodological limitations in our study. One was the use of proximity to agricultural fields as an independent variable in this study. This use assumes that there is a direct correlation between agricultural proximity and pesticide exposure, and that this correlation is fairly uniform. Due to the use of this imperfect characteristic as an independent variable, there is a slight level of uncertainty to how exactly agricultural proximity correlates with neurological outcomes. However, this uncertainty can be subsided with previous research conducted on agricultural proximity and pesticide exposure. As cited in the introduction, many studies have found a linear regression between levels of pesticide exposure and proximity to agricultural fields. In other words, previous studies have established that greater residential distance from agricultural fields is correlated with lower levels of pesticide exposure and vice versa. These established relationships serve to decrease uncertainty within our study, by showing that we can correlate closeness to agricultural fields with pesticide exposure. Furthermore, our choice of using proximity as an independent variable rather than exact pesticide exposure is a fairly novel one. It allows for increased recognition of the implications of living near agricultural areas in a cost-effective manner. This method of study may additionally increase political relevance and action on this matter.

Lack of knowledge on the exact pesticides used is another weakness of the study. Knowledge of the exact pesticides used would have allowed for a more thorough investigation of the neurological outcomes, as different pesticides have different effects on the body. However, through interviews with the storeowners in the communities we were able to compile a list of pesticides that sold in the surrounding areas. It is quite likely that the pesticides used by the workers were from this list (Table 2). In the future, we plan to investigate this further so that we may pinpoint the neurological outcomes to specific pesticides.

Additionally, this study was a cross-sectional one and this itself poses a limitation. This structure of study hinders the inferences we can make about the cause and effect relationship. As it is a cross sectional study, we cannot prove that neurological outcomes are caused by agricultural proximity or vice versa. However, we argue that in this case, it is likely that agricultural proximity caused the neurological outcomes; as it is very unlikely that the neurological outcomes caused agricultural proximity. There may be a confounding variable that caused this relationship, but at this time, we have not identified any.

This study was conducted in rural Bangladesh and the majority of families included in the study live in marginal economic conditions. Therefore, the results may not be generalizable across all population groups; they likely will only apply to communities with similar rural sociodemographic characteristics.

## 5. Conclusions

We studied several neuro-psychological parameters (i.e., vibrotactile threshold, dominant ulnar NCV, trail making test and depression assessment by CES-D) in non-farmer residents living within 200 m or closer vs. far from farming lands where pesticides are used extensively via a cross-sectional analysis. Our results showed that people living in close proximity to agricultural fields demonstrated negative neurological outcomes in spite of not being directly involved in agricultural activities or active handling of pesticides when compared with people living more than 200 m from the fields. Therefore, policy makers need to put emphasis on developing mitigation plans for residences near farming lands to prevent passive pesticide exposure. A prospective study with a larger sample size is recommended for future epidemiological studies to obtain conclusive evidence regarding the relationship between the proximity to pesticide-exposed fields and its neuropsychiatric effects on residents living nearby.

## Figures and Tables

**Table 1:** Sample sociodemographic characteristics (*n* = 57).

Variables	Far Away from Ag Field (*n* = 19) (≥200 m) Mean ± SD or *N* (%)	Close Proximity to Ag Field (<200 m) (*n* = 38) Mean ± SD or *N* (%)	*p*-Value for Group Difference
Age (Years)	42.32 ± 6.26	40.53 ± 6.87	0.33
Gender (Male)	35 (92.1)	18 (94.7)	0.71
Income (Thousands of Bangladeshi Taka per year)	106.74 ± 35.88	131.26 ± 66.70	0.08
Land ownership (Acres)	2.92 ± 2.00	2.95 ± 2.09	0.96
Number of children in immediate household	2.37 ± 1.32	2.58 ± 1.50	0.59
Number of adults in immediate household	2.97 ± 1.08	3.16 ± 1.95	0.64
BMI (kg/m^2^)	21.80 ± 3.23	26.89 ± 5.34	0.001
Educational qualification			0.10
Primary and below	24 (63.2)	11 (57.9)	
Secondary and above	14 (36.8)	8 (42.1)	
Marital status (Married)	34 (89.5)	19 (100)	0.34
Major occupation			
Small business	10 (26.3)	8 (42.1)	0.06
Day labor	17 (44.7)	2 (10.5)	
Other	11 (29.9)	9 (46.4)	

**Table 2:** Types of pesticides used by farmers in Matlab study area, Bangladesh.

Chemical Type	Trade Name	WHO Categorized Class	WHO Hazardous Characteristics
Carbamate	Carbofuran	Class Ib	Highly hazardous
Organophosphate	Chlorpyrios	Class II	Moderately hazardous
Organophosphate	Diazinon	Class II	Moderately hazardous
Oxadiazon	Ronstar	Class U	Unlikely to present acute hazard in normal use
Pyrethroid	Cypermethrin	Class II	Moderately hazardous
Zeta-cypermethrin	Ostad	Class Ib	Highly hazardous

**Table 3:** Association of proximity of agricultural (Ag) field with neurological, neurobehavioral and mental health outcomes.

Variables	Unadjusted Mean Mean (95% CI)		GLM Unadjusted Model *p*-Value	Adjusted Mean * Mean (95% CI)		GLM Adjusted Model *p*-Value
	Far Away from Ag Field (*n* = 19)	Close Proximity to Ag Field (*n* = 38)		Far Away from Ag Field (*n* = 19)	Close Proximity to Ag Field (*n* = 38)	
***Neurological and Neurobehavioral Outcomes***						
Vibrotactile threshold score (Right big toe)	3.10 (2.75, 3.44)	3.60 (3.36, 3.84)	0.02	3.09 (2.70, 3.49)	3.61 (3.34, 3.87)	0.05
Vibrotactile threshold score (Left big toe)	2.87 (2.51, 3.24)	3.63 (3.36, 3.89)	0.002	2.83 (2.40, 3.25)	3.65 (3.37, 3.93)	0.004
Nerve conduction velocity (m/s)	56.63 (53.69, 59.57)	59.23 (57.16, 61.32)	0.14	56.37 (53.10, 59.64)	59.37 (57.19, 61.55)	0.16
Trail making test A (sec)	56.47 (42.76, 70.18)	76.73 (66.78, 86.69)	0.02	61.02 (47.10, 74.95)	74.46 (64.77, 84.16)	0.12
Trail making test B ^#^ (sec)	108.68 (91.32, 126.04)	137.29 (124.50, 150.10)	0.01	110.80 (92.65, 128.94)	136.13 (122.92, 149.34)	0.004
Self-reported behavioral symptoms score	3.31 (2.29, 4.33)	3.11 (2.38, 3.82)	0.73	3.35 (2.25, 4.46)	1.08 (2.34, 3.81)	0.70
Self-reported cognitive symptoms score	1.78 (0.97, 2.60)	1.39 (0.81, 1.97)	0.43	2.18 (1.26, 3.12)	1.20 (0.58, 1.81)	0.11
Self-reported sensory symptoms score	1.90 (0.69, 3.10)	2.66 (1.80, 3.51)	0.30	1.96 (0.74, 3.16)	2.62 (1.82, 3.43)	0.39
Self-reported motor symptoms score	1.30 (0.60, 2.03)	1.60 (1.10, 2.11)	0.50	1.49 (0.70, 2.34)	1.51 (0.95, 2.04)	0.98
Self-reported total symptoms score	10.42 (7.54, 13.29)	10.65 (8.62, 12.70)	0.89	11.22 (8.22, 14.22)	10.25 (12.25)	0.61
***Mental Health Outcomes***						
CES-D depression score	2.68 (1.57, 3.79)	2.19 (1.40, 2.97)	0.47	3.20 (2.00, 4.45)	1.99 (1.09, 2.72)	0.10
CES-D well-being Score	0.90 (0.08, 1.71)	1.03 (0.44, 1.60)	0.79	1.01 (0.16, 2.04)	0.93 (0.29, 1.54)	0.76
CES-D somatic score	6.21 (5.12, 7.30)	6.55 (5.78, 7.32)	0.60	7.01 (5.85, 8.20)	6.15 (5.36, 6.93)	0.26
CES-D interpersonal score	0.11 (-0.22, 0.44)	0.45 (0.21, 0.68)	0.11	0.10 (−0.29, 0.49)	0.45 (0.19, 0.71)	0.17
CES-D total score	9.90 (7.38, 12.40)	10.21 (8.43, 11.98)	0.83	11.45 (8.71, 14.20)	9.43 (7.60, 11.26)	0.26

* Analyses adjusted for age, BMI, educational qualification and yearly household income. ^#^ 18 subjects from high proximity of Ag field and 35 subjects from close proximity to Ag field groups completed the trail making test B. GLM: Generalized Linear Models.
